# Effects of *Phyllanthus reticulatus* on lipid profile and oxidative stress in hypercholesterolemic albino rats

**DOI:** 10.4103/0253-7613.71923

**Published:** 2010-12

**Authors:** V. Maruthappan, K. Sakthi Shree

**Affiliations:** Department of Zoology, Unit of Endocrinology, Bharathiar Univeristy, Coimbatore - 641 046, India; 1 PG and Research Center, Government Arts College (Autonomous), Coimbatore - 641 018, India

**Keywords:** Hypercholesterolemia, lipid profile, oxidative stress, *P. reticulatus*

## Abstract

**Objective:**

This study was designed to investigate the effect of *Phyllanthus reticulatus* on lipid profile and oxidative stress in hypercholesterolemic albino rats.

**Materials and Methods:**

Hypercholesterolemia was induced in albino rats by administration of atherogenic diet for 2 weeks. Experimental rats were divided into different groups: normal, hypercholesterolemic control and *P. reticulatus* treated (250 and 500 mg/kg body weight doses for 45 days). After the treatment period of 45^th^ day triglyceride, VLDL-cholesterol, HDL-cholesterol, total cholesterol (TC), LDL-cholesterol and oxidative stress (protein carbonyl) were assayed and compared with hypercholesterolemic control.

**Results:**

The aqueous extract of *P. reticulatus* (250 mg and 500 mg/kg) produced significant reduction (*P* < 0.05) in triglyceride, VLDL-cholesterol, total cholesterol (TC), LDL-cholesterol and oxidative stress (protein carbonyl) while increased HDL-cholesterol in atherogenic diet-induced hypercholesterolemic rats at the end of the treatment period (45 days). However, the reduction in the above parameters was comparable with hypercholesterolemic control. Thus, aqueous extract of *P. reticulatus* is effective in controlling TC, lipid profile and oxidative stress in hypercholesterolemic animals.

**Conclusion:**

The results suggest the aqueous extract of *P. reticulatus* can be utilized for prevention of atherosclerosis in hypercholesterolemic patients.

## Introduction

Hypercholesterolemia is associated with serious health risks and increased mortality. Hypertension, hyperlipidemia, insulin resistance and glucose intolerance are known as cardiac risk factors that cluster in obese individuals.[1] The persistence of hypercholesterolemic state causes enhanced oxidative stress, leading to the development of atherosclerosis, coronary artery disease (CAD) and other complications of obesity.[2,3]

In the recent years, there is growing interest in herbal medicines all over the world. Traditional medicinal plants having antilipidemic property can prove to be an useful source for the development of new oral hypolipidemic agents or simple dietary adjuvant to existing therapies. Ethnopharmacological surveys indicate that more than 1200 plants are used in traditional medicine for their hypolipidemic activity.[4,5] The hypolipidemic activity of a number of plants/plant products has been evaluated and confirmed in animal models,[6,7] as well as in human beings.[8,9]

*Phyllanthus reticulatus* (Family—Euphorbiaceae) is a climbing shrub which grows all over India.[10] It has been shown to have hypotensive effects and its folklore use in gastric complaints including colic and constipation has been reported.[11] *Phyllanthus niruri* has been shown to possess significant hepatoprotective activity in fatal liver diseases such as liver cirrhosis and hepatocellular carcinoma.[12] Chemical studies demonstrated the presence of octacosanol, teraxerol acetate, friedeline, teraxerone, betulin, sitosterol, etc.[13] Although *P. reticulatus* has not been studied for significant chemical as well as biological studies, the plants of this genus were reported to contain lignins, flavonoids, triterpenoids, alkaloids and polyphenolic compounds.[14] Flavonoids and polyphenolic compounds have a potential role in the prevention of various diseases through their antioxidant activity.[15] The present work was designed to study the lipid profile and oxidative stress of the aqueous extract of the aerial parts of *P. reticulatus* in rat model.

## Materials and Methods

### 

#### Animals

Adult albino rats (Wistar strain) weighing 180–200 g of either sex were used for this study. The animals were housed in polypropylene cages at controlled temperature (26 ± 2°C), relative humidity (60 ± 5%) with a 12–12 h light–dark cycle. The rats were fed with standard laboratory diet and water was provided *ad libitum*. The animals were maintained as per the CPCSEA guidelines and regulations and the study was approved by the Institutional Animals Ethics Committee at Bharathiar University, Coimbatore, India. The composition of atherogenic diet used during the study was given as in Table 1.

**Table 1:** Composition of normal and atherogenic diet

*Composition*	*Normal diet (%)*	*Atherogenic diet (%)*
Protein (milk powder)	12	10
Carbohydrates (wheat flour)	71	61
Sugar	05	05
Fat (butter)	05	16
Salts	04	04
Vitamins	01	02
Fibers	02	01
Cholesterol	-	01
Total weight	100 g	100 g

#### Preparation of the extract

Aerial parts (stem and leaves) of *P. reticulatus* were collected in July–September from Maruthamalai Hill, Coimbatore (India). The plant was identified and authenticated with the help of Botanical Survey of India, Coimbatore (India). The plant material was dried under shade and powdered in a grinder. The powdered material (100 g) was extracted with double-distilled water by the hot continuous percolation method in a Soxhlet apparatus. The extract was evaporated to dryness under vacuum and dried in a vacuum desiccator to obtain a residue of 15.65 g.

#### Induction of experimental hypercholesterolemia

In order to induce hypercholesterolemia, the method reported by Bopanna *et al*. was followed.[16] The animals were divided into four groups of six rats each and they received the following diets with or without treatment for 45 days orally:

Group I: Normal diet.
Group II: Atherogenic diet containing 1% cholesterol.
Croup III: Atherogenic diet + aqueous extract of *P. reticulatus* (250 mg/kg b.w.).
Group IV: Atherogenic diet + aqueous extract of *P. reticulatus* (500 mg/kg b.w.).

At the end of the treatment, the rats were fasted overnight and killed by decapitation. Blood was collected and serum was separated and stored in a refrigerator (4°C) until assay.

#### Measurement of serum lipid profile

Total cholesterol (TC),[17] serum triglyceride,[18] high density lipoprotein (HDL),[19] and protein carbonyl levels,[20] were measured while very low density lipoprotein (VLDL) was calculated as triglyceride/5 and low density lipoprotein (LDL) was calculated using the equation: LDL = total cholesterol – (HDL + VLDL). The atherogenic index was calculated using the following formula:

$$\mathrm{Atherogenic}\;\mathrm{Index}\;=\;\frac{\mathrm{Serum}\;\mathrm{cholesterol}}{\mathrm{Serum}\;\mathrm{HDL}\;–\;\mathrm{cholesterol}}$$

#### Drugs and chemicals

All the chemicals were used of analytical grade, obtained from M/s. SISCO Research Laboratories Pvt. Ltd, Mumbai.

#### Statistical analysis

Statistical analysis was carried out using Student’s *t*-test.[21]

## Results

Phytochemical screening revealed the presence of lignins, flavonoids, triterpenoids, alkaloids, polyphenolic compounds and mucilage in the aqueous extract of *P. reticulatus*. Feeding of atherogenic diet increased serum cholesterol, triglyceride, LDL-cholesterol and HDL-cholesterol level, VLDL-cholesterol and protein carbonyl level when compared to normal group at over a period of 45 days. Administration of aqueous extract of *P. reticulatus* (250 and 500 mg/kg per day) showed statistically significant decrease in total cholesterol (*P* < 0.05), triglyceride (*P* < 0.001), LDL-cholesterol (*P* < 0.05), VLDL-cholesterol (*P* < 0.001) and protein carbonyl level (*P* < 0.05) while increase in HDL-cholesterol level (*P* < 0.05) as compared to hypercholesterolemic animals [Table 2]. The aqueous extract treated animals showed decrease in the atherogenic index and increased percentage of protection at both the doses, i.e. 250 and 500 mg/kg [Table 3].

**Table 2:** Effect aqueous extract of *P. reticulatus* on serum lipid profile in normal and atherogenic diet-induced hypercholesterolemic rats

*Groups*	*Total cholesterol (mg/dL)*	*Triglyceride (mg/dL)*	*VLDL (mg/dL)*	*HDL (mg/dL)*	*LDL (mg/dL)*	*Protein carbonyl (nmol/mg prot)*
Group I (Normal)	160.0 ± 14.4	176 ± 3.0	38 ± 1.6	46 ± 1.4	50.41 ± 2.96	0.120 ± 0.40
Group II (Atherogenic diet only)	240.0 ± 16.0	236 ± 3.6	49 ± 1.8	51 ± 1.02	55.81 ± 0.46	0.200 ± 1.20
Group III (Atherogenic diet + aqueous extract, 250 mg/kg)	139.1 ± 34.8**	200 ± 3.31**	42 ± 1.12*	59 ± 1.76 **	52.89 ± 0.98*	0.136 ± 0.25*
Group IV (Atherogenic diet + aqueous extract, 500 mg/kg)	130.2 ± 30.0**	186 ± 3.21**	39 ± 1.16**	63 ± 1.89**	51.41 ± 0.38*	0.130 ± 0.15*

Statistical significance in comparison to groups III, IV with group II;

* *P* < 0.05

** *P* < 0.001

**Table 3:** Atherogenic index in various study groups

Groups	Atherogenic index	Protection* (%)
Group I (Normal)	2.163	
Group II (control)	3.200	----
(Atherogenic diet only) Group III (Atherogenic diet + aqueous extract 250 mg/kg)	1.807	44.51
Group IV (Atherogenic diet + aqueous extract 500 mg/kg)	1.225	67.82

* $$\mathrm{Protection}\;(\%)=\frac{\mathrm{Atherogenic}\;\mathrm{index}\;\mathrm{of}\;\mathrm{control}\;–\;\mathrm{atherogenic}\;\mathrm{index}\;\mathrm{treated}\;\mathrm{group}\;}{\mathrm{Atherogenic}\;\mathrm{index}\;\mathrm{of}\;\mathrm{control}}\times 100$$

## Discussion

The effect of *P. reticulatus* on lipid profile and oxidative stress in hypercholesterolemic albino rats was evaluated in this study. *P. reticulatus*, an Indian herbal plant, possesses cardioprotective and lipid lowering properties. Treatment with *P. reticulatus* extract produced a significant decrease in the serum lipid level in atherogenic diet-induced hypercholesterolemia in rats. Maruthappan and Sakthi Shree[22,23] found that the feeding of *Adenanthera pavonina* and *Terminalia chebula* lowered the total cholesterol and its fractions in lipoproteins. They reported hypolipidemic activity of the saponins, flavonoids and polyphenolic compounds from *A. pavonina*. Beta-sitosterol, a phytosterol, is reported as useful in the treatment of hyperlipidemia.[16] Sudhessh *et al*.[24] reported that condensed tannins of *Solanum melongena* are reduced in hyperlipidemia. The aqueous extract of *P. reticulatus* contains lignins, flavonoids, triterpenoids, alkaloids, polyphenolic compounds and mucilage. The high amount of lignins, flavonoids present in *P. reticulatus* may be responsible for the hypocholesterolemic effect. Use of diet rich in saturated fats and an increase in coronary heart disorder (CHD) has been observed in the developing countries for the past few decades.[25]

Pretreatment of *P. reticulatus* reduced the atherogenic diet-induced hypocholesterol manifestations in multiple ways. Increase in the level of serum triglyceride, total cholesterol, LDL-cholesterol and VLDL-cholesterol in the *P. reticulatus*-treated group indicate that phytochemicals may be interfering with metabolism or biosynthesis of lipids. Pretreatment with *P. reticulatus* showed reduction in serum lipid levels with concomitant increase in HDL-cholesterol. Decrease in serum lipid profiles and increase in HDL-cholesterol in *P. reticulatus*-treated group may be due to the present of phytochemicals. Lipid lowering effect of *P. reticulatus* could be due to inhibition of hepatic cholesterol biosynthesis, increased fecal bile acid secretion and stimulation of receptor-mediated catabolism of LDL-cholesterol and increase in uptake of LDL from blood by liver.[26] The atherogenic index (TG/HDL-C ratio) used to predict risk of CHD and marker of small, dense LDL-C (an atherogenic lipoprotein)[27,28] were significantly reduced by the *P. reticulatus* extract, indicating the beneficial effect of extract in cardiovascular diseases.

Oxidative stress is an important event in the development and maintenance of atherosclerosis.[29] Protein carbonyl is the most widely used biomarker for oxidative damage to proteins by multiple forms reactive oxygen species (ROS).[30] On the basis of the measurement of protein carbonyl, it has been suggested that the accumulation of oxidized proteins is associated with atherosclerosis.[31] However, the relationship between serum lipid levels and protein carbonylation is not clear.

## Conclusion

In this study, an increase in serum HDL-cholesterol with a concomitant decrease in other lipids was observed. It can be concluded from the present data that the levels of total cholesterol, triglyceride, LDL-cholesterol, VLDL-cholesterol and protein carbonyl which are raised in atherogenic diet are lowered significantly with the aqueous extract of *P. reticulatus*. Aqueous extract of *P. reticulatus* can be utilized for prevention of atherosclerosis in hypercholesterolemic patients.

## References

[1] Anuradha CV, Ravikumar P (2001). Restoration on tissue antioxidants by fenugreek seeds (Trigonella Foenum Graecum) in alloxan-diabetic rats. Ind J Physiol Pharmacol.

[2] Hunt JV, Smith CC, Wolff SP (1990). Autoxidation glycosylation and possible involvement of peroxides and free radicals in LDL modification by glucose. Diabetes.

[3] Oberly LW (1988). Free radicals and diabetes. Free Radi Biol Med.

[4] Grover JK, Yadav S, Vats V (2002). Medicinal plants of India with anti-diabetic potential. J Ethnopharmacol.

[5] Li WL, Zheng HC, Bukuru J, De Kimpe N (2004). Natural medicines used in the traditional Chinese medical system for therapy of diabetes mellitus. J Ethnopharmacol.

[6] Gupta RK, Kesari AN, Watal G, Murthy PS, Chandra R, Tandon V (2005). Nutritional and Hypoglycemic Effect of Fruit pulp of *Annona squamosa* in Normal Healthy and Alloxan-Induced Diabetic Rabbits. Annals Nutri Meta.

[7] Kesari AK, Gupta RK, Singh SK, Diwakar S, Watal G (2006). Hypogycemic and antihyperglycemic activity of *Aegle marmelos* Seed Extract in Normal and Diabetic Rats. J Ethnopharmacol.

[8] Herrera-Arellano A, Aguilar-Santamaria L, Garcia-Hernandez B, Nicasio-Torres P, Tortoriello J (2004). Clinical trial of *Cecropia obtusifolia* and *Marrubium vulgare* leaf extracts on blood glucose and serum lipids in type 2 diabetics. Phytomedicine.

[9] Jayawardena MH, De Alwis NM, Hettigoda V, Fernando DJ (2005). A double blind randomized placebo controlled cross over study of herbal preparation containing *Salacia reticulata* in the treatment of type 2 diabetes. J Ethnopharmacol.

[10] Ghani A (2003). Medicinal Plants of Bangladesh, Chemical constituents and uses. Asiatic Society of Bangladesh.

[11] Wells BG, Dipiro JT, Schwinghammer, TL, Hamilton CW (2003). Pharmacotherapy Handbook.

[12] Gneti KR, Agarwal S (1995). Hepatoprotective studies on *Phyllanthus niruri* and quercetin. J Exptl Biol.

[13] Rates SM (2001). Plants as source of drugs. Toxicon.

[14] Yoshida T, Seno K, Takama Y, Okanda T (1964). Tannins and related polyphenol of Euphorbiaceous plants. Phytochem.

[15] Kaur G, Alam MS, Jabbar Z, Javed K, Athar M (2006). Evaluation of antioxidant activity of *Cassia siamea* flowers. J Ethnopharmacol.

[16] Bopanna KN, Bhagyalakshmi N, Rathod SP, Balaraman R, Kannan J (1997). Cell culture derived *Hemidesmus indicus* in the prevention of hypercholesterolemia in normal and hyperlipidemic rats. Ind J Pharmacol.

[17] Roeschlau P, Bernt E, Gruber W (1974). Enzymatic determination of total cholesterol in serum. Z Klin Chem Klin Biochem.

[18] Buccolo G, David H (1973). Quantitative determination of serum triglycerides by the use of enzymes. Clin Chem.

[19] Burstein M, Scholnick HR, Morfin R (1970). Rapid method for the isolation of lipoprotein from human serum by precipitation with polyanions. J Lipid Res.

[20] Levine RL, Garland D, Oliver CN, Amici A, Climent I, Lens AG (1990). Determination of Carbonyl content in oxidatively modified proteins. Methods Enzymol.

[21] Bennet CA, Franklin NL (1967). Statistical analysis in chemistry and chemical industry.

[22] Maruthappan V, Sakthi Shree K (2010). Hypolipidemic activity of Haritaki (Terminalia Chebula) in atherogenic diet induced hyperlipidemic rats. J Adv Pharm Tech Res.

[23] Maruthappan V, Sakthi Shree K (2010). Blood cholesterol lowering effect of *Adenanthera pavonina* seed extraction atherogenic diet induced hyperlipidemia rats. Int J Pharma Sci Res.

[24] Sudhessh S, Vijay KS, Sandhya C, Vijayalakshmi NR (1996). Toxic effects of condensed tannins from *Solanum melongena* on rats. J Ecotoxicol Environ Monit.

[25] Kulkarni SK, Gurpreet Kaur (1999). Obesity: An insight into its neurochemical basis and treatment. Ind J Pharmaco.

[26] Khanna AK, Ramesh C, Kapoor NK (1996). Terminalia arjuna: An Ayurvedic cardiotonic regulates lipid metabolism in hyperlipidemic rats. Phytother Res.

[27] Hanak V, Munoz J, Teague J, Stanley A, Bittner V (2004). Accuracy of the triglyceride to high-density lipoprotein cholesterol ratio for prediction of the low-density lipoprotein phenotype B. Am J Cardiol.

[28] Packard C, Caslake M, Shepherd J (2000). The role of small, dense low density lipoprotein (LDL): A new look. Int J Cardiol.

[29] Madamanchi NR, Vendrov A, Runge MS (2005). Oxidative stress and vascular disease. Arterioscler Thromb Vasc Biol.

[30] Dalle-Donne I, Rossi R, Colombo R, Giustarini D, Milzani A (2006). Biomarkers of oxidative damage in human disease. Clin Chem.

[31] Stadtman ER, Levine RL (2003). Free radical-mediated oxidation of free amino acids and amino acid residues in proteins. Amino Acids.

